# Functional Dissection of Auxin Response Factors in Regulating Tomato Leaf Shape Development

**DOI:** 10.3389/fpls.2018.00957

**Published:** 2018-07-04

**Authors:** Lingjie Wu, Zhendong Tian, Junhong Zhang

**Affiliations:** Key Laboratory of Horticultural Plant Biology, Ministry of Education, Huazhong Agricultural University, Wuhan, China

**Keywords:** auxin, development, leaf shape, *SlARFs*, *SlIAA9*, tomato

## Abstract

The phytohormone auxin is involved in many aspects of plant growth and developmental processes. The tomato Aux/IAA transcription factor SlIAA9/ENTIRE/E plays an important role in leaf morphogenesis and fruit development, and the *E* gene encodes a protein from the Aux/IAA family of auxin response repressors. Both *SlIAA9*-RNAi transgenic and *entire* (*e*) mutant plants reduce the leaf complexity in tomato, but the underlying mechanism is not yet completely resolved. Auxin signaling is known to regulate target genes expression via Aux/IAA and ARFs (auxin response factors) transcriptional regulators. ARFs mediate a wide range of developmental processes. Through an Y2H (yeast two-hybrid) assay coupled with expression profiling of the *SlARF* genes family, we identified a group of ARFs: SlARF6A, SlARF8A, SlARF8B, and SlARF24. Pull-down and BiFC (Bimolecular Fluorescence Complementation) results demonstrated that these SlARFs interact with SlIAA9 *in vitro* and *in vivo*, and the *e* mutation altered the expression patterns of multiple *SlARFs*. The simple leaves of the *e* mutant were partially converted to wild-type compound leaves by VIGS (virus-induced gene silencing) of these four *SlARFs*. Furthermore, IAA content in these samples was significantly increased compared to the *e* mutant. In addition, SlARF6A and SlARF24 bound to the *SlPIN1* promoter and act as transcriptional activators to regulate genes expression involved in leaflet initiation. It may also suggest that SlARFs regulate leaf morphology through direct binding to auxin-responsive genes in the absence of SlIAA9, providing an insight for the role of *SlARFs* in leaf shape development.

## Highlights

We firstly found that SlARF6A, SlARF8A, SlARF8B, and SlARF24 could regulate tomato leaf development in a redundant manner; Furthermore, SlARF6A and SlARF24 bound to the *SlPIN1* promoter to regulate genes expression involved in leaflet initiation.

## Introduction

Leaves are one of the main organs of flowering plants, and exhibit a tremendous diversity in shape and size. The shape of leaves varies enormously within the same species and individual plants, and can be ascribed to ranging from simple to compound. Variation is one of the most conspicuous aspects of plant diversity in leaf shape. This diversity is often achieved by the adjustment of leaf blade dissection to form lobes or leaflets (Ben-Gera et al., 2012). Simple leaves comprise of a single continuous blade, whereas compound leaves are composed of multiple discontinuous blade units termed as leaflets (Koenig et al., 2009). Leaves are formed at the flanks of the shoot apical meristem (SAM). Following the initiation of new shoot morphogenesis, leaves establish the basic framework for shape and size. Subsequently, organogenesis of lateral appendages occurs through differentiation and expansion of leaf tissue (Shwartz et al., 2016). Furthermore, the wild type leaves consist of primary, secondary, and intercalary leaflets with lobed margins in tomato (Berger et al., 2009).

After the formation and differentiation of the leaf primordia in the SAM, the development of the leaf primordium occurs (Merelo et al., 2016). Previous studies have discovered that two mechanisms are involved in the development of the leaf primordia. The first occurs through mutual repression between KNOX proteins and ARP (AS1/RS2/PHAN) MYB-domain proteins (Waites et al., 1998; Tsiantis et al., 1999; Byrne et al., 2000; Ori et al., 2000). The second mechanism demonstrates that the formation of leaf delimitation is controlled by *PINOID* (*PID*) and auxin efflux carrier *PIN-FORMED1* (*PIN1*) which mediates local auxin accumulation (Furutani et al., 2004). In leaf development, AS1 represses the expression of *KNOX* gene *BP (BREVIPEDICELLUS)*, while the function of the local auxin maxima alongside AS1 remains partly dependent on *BP* regulation. The *SHOOT MERISTEMLESS* (*STM*) gene is needed for SAM formation and maintenance, which prevents *AS1* gene expression in the meristem (Byrne et al., 2000). In addition, auxin activities and KNOX proteins might form a feedback loop to facilitate leaf meristem delimitation (Zgurski et al., 2005; Hay et al., 2006).

Studies have indicated that leaf development is coordinated by a cross-talk between different hormones (Shwartz et al., 2016) with auxin playing a crucial role. The precise distribution and location of auxin signaling regulates proper leaf development in a specific spatiotemporal developmental context (Bilsborough et al., 2011). The auxin maxima is concentrated on the leaflet initiation area in the development of tomato leaves, resulting in lamina growth patterns (Ben-Gera et al., 2016). Auxin acts as an inducer of organogenesis. There are postulated inhibitory fields around existing primordia which are thought to result from low concentrations of auxin (Reinhardt et al., 2003; de Reuille et al., 2006; Jonsson et al., 2006). Endogenous auxin levels and localization are altered in developing leaves leads to leaf simplification phenotypes (Shwartz et al., 2016). *SlIAA9* is an auxin regulator belonging to the *Aux/IAA* transcription factors gene family. Not only do *SlIAA9*-RNAi plants display simple leaves and parthenocarpy instead of compound leaves and seeded fruit characteristic typically seen from wild type (AC), but also the silenced plants have auxin-related growth alterations (Wang et al., 2005). The adjustment of *Aux/IAA* and *SlARF* genes and the downregulation of MADS box genes mediate fruiting, and early fruit development is regulated by the regulatory and metabolic events both in the absence and presence of pollination/fertilization (Wang et al., 2009). Meanwhile, tomato *e* mutant is reported as a single-base deletion in the coding region of the *SlIAA9* gene and exhibits single based lamina with primary leaves partially fused (Zhang et al., 2007), *E* mRNA is discovered throughout the leaf margin (Koenig et al., 2009). Thus, *SlIAA9* plays a role in limiting lamina growth between developing leaflets by locally inhibiting auxin responses. *GOB* (*GOBLET*) encodes a NAC-domain transcription factor and its expression is intact in the simplified leaves of *entire* (*e*) mutants in tomato. Leaves of single *gob* or *e* mutants formed only primary leaflets, and downregulation of both *GOB* and *E* (*SlIAA9*) contributed to the complete abolishment of leaflet initiation. This indicates those auxin response and leaflet morphogenesis are modulated by GOB and E via partly redundant pathways (Blein et al., 2008; Ben-Gera et al., 2012). The tomato *clau* (*clausa*) mutant exhibits elaborate compound leaves. CLAU might negatively regulate the expression of *GOB*, and *GOB* expression is up-regulated in the compound leaf mutant *lyr* (*lyrate*). However, the enhancement of the *clau* phenotype by *lyr* indicates that *clau* and *lyr* affect *GOB* and leaf development in different pathways (Bar et al., 2015). Higher expression of *LA* (*LANCEOLATE*) during the early stages of leaf development result in a simpler leaf shape, likely regulated in part by gibberellic acid (GA) levels (Yanai et al., 2011). The expression of *TKn1* in the leaf primordium is needed for compound structure formation (Hareven et al., 1996). *miR164* negatively regulates *GOB*-like genes, and leaf-specific overexpression of *miR164* induces a loss of secondary leaflet initiation and smooth leaflet margins (Berger et al., 2009). The miR160 targets a group of ARFs which antagonize lamina growth and auxin response in conjunction with E plants. Leaflet separation is assured by different type of auxin signal antagonists (Ben-Gera et al., 2016). Auxin, E, GOB, LYR, and mir160-targeted ARFs collaborate to specify leaflet initiation and promote leaflet separation (Kimura et al., 2008). However, the underlying molecular mechanism of how functional redundancies among SlARF proteins regulate leaf shape development in tomato remains an open question.

Aux/IAA protein can repress ARF transcription factors via protein to protein interaction (Tiwari et al., 2001), and the degradation of Aux/IAA proteins can relieve ARF proteins for auxin-responsive gene transcription (Tan et al., 2007). SlIAA9 protein mediates leaf morphogenesis by participating in auxin signal transduction (Wang et al., 2005; Goetz et al., 2007). Furthermore, ARFs represent essential factors in the transduction of auxin signaling, and multiple ARFs were previously shown to interact with IAA9 (Korasick et al., 2014; Piya et al., 2014). In this work, we showed that tomato plants with a silenced SlIAA9 complex change from simple to complex leaf morphology, providing an insight for the significant role of functional redundancies among SlARF proteins in leaf morphogenesis. This indicates that SlARFs may mediate phenotypic plasticity in foliar organogenesis, and that further studies on SlARFs may reveal insights into the evolution of plant leaves.

## Materials and Methods

### Plant Materials and Growth Conditions

Tomato plants (*Solanum lycopersicum* cv. Ailsa Craig) and *e* mutants in the Ailsa Craig background were grown under standard greenhouse conditions (14 h day/10 h night cycle, 25/20°C day/night temperature, 60–75% relative humidity). The *e* mutation plants prepared for VIGS assay were kept in a growth chamber (16 h day/8 h night cycle, 20–22°C, 50% relative humidity).

### qRT-PCR

Total RNA from all samples was isolated using the TRIzol reagent (Invitrogen, United States). The RNA was treated with DNase I at 37°C for 30 min to remove residual genomic DNA. Using the HiScript II 1st cDNA Synthesis Kit (Vazyme, China) to synthesis the first-strand cDNA according to the manuscript’s protocol. The cDNA concentrations were normalized according to actin expression levels for qRT-PCR analysis. qRT-PCRs were performed using the power SYBR Premix Ex Taq kit and the TaKaRa two-step method (TaKaRa, Japan). PCR products were quantified using the Roche Light Cycler 480 Real-Time PCR Detection System and the SYBR Green I Master Kit (Roche, Switzerland). The PCR program was as follows: 95°C for 45 s; 40 cycles of 95°C for 10 s, 58°C for 25 s, and 72°C for 20 s. For all qRT-PCR experiments, at least three biological replicates were performed, and each reaction was run in triplicate.

### Yeast Two-Hybrid Assay (Y2H)

For yeast two-hybrid assay, the full-length coding sequence of each *SlARF* (Supplementary Table S1) and *SlIAA9* (Solyc04g076850) were cloned from various tissues of Ailsa Craig. Recombinant plasmid pGBKT7-SlIAA9 and pGADT7-SlARF were constructed by inserting *SlIAA9* and *SlARF* into pGBKT7 and pGADT7 vectors separately. The two plasmids were co-transformed into the yeast strain AH109 by small-scale yeast transformation method. The transformants grew on the SD/-Trp/-Leu drop-out medium. After colony formation, transformants were transferred to SD/-Leu/-Trp/-His/-Ade drop-out medium with 40 μg ml^-1^ X-gal.

### Bimolecular Fluorescence Complementation (BiFC) Assays

The *SlIAA9* ORF without the stop codon was constructed using the *pUC-SPYNE/pSPYNE-35S* vector to produce SlIAA9-YFP^N^ fusions, and the SlARF ORFs without the stop codon were cloned into the *pUC-SPYCE/pSPYCE-35S* vector to generate SlARFs-YFP^C^ fusion proteins. Protoplasts were extracted from 1-week-old *Arabidopsis* Col-0 suspension cell culture and the corresponding constructs were co-transformed into them. The transfected protoplasts were assayed for fluorescence after 12–18 h of expression. All primer sequences used in this analysis are listed in Supplementary Table S2.

### *In Vitro* Pull-Down Assay

The recombinant plasmids were transformed into BL21(DE3)pLysS chemically competent cells. SlIAA9-GST was purified with Glutathione Agarose (Thermo Fisher Scientific, United States) according to the company manual instruction. MBP, MBP-SlARF6A, MBP-SlARF8A, MBP-SlARF8B, and MBP-SlARF24 were purified as fusion proteins immobilized with amylose resin (New England Biolabs, United States) following standard protocols. Five micrograms of GST-SlIAA9 protein were pre-incubated with 10 μL pre-washed amylose resin in 150 μL incubation buffer (1 mM NaCl, 20 mM MgCl_2_, 0.2% Triton X-100, and 0.1 M HEPES at pH7.2) for 1 h at 4°C. The resin was collected by centrifugation and washed five times with washing buffer (20 mM Tris-HCl at pH 7.5, 300 mM NaCl, 0.1 mM EDTA, 0.5% Triton-X100). The pull-down proteins were detected by western blot with an α-GST antibody (Thermo Fisher Scientific, United States). All primer sequences used in this analysis are listed in Supplementary Table S2.

### VIGS Assays

To generate the VIGS constructs, 208 bp, 157 bp, and 238 bp fragments of the gene *SlARF6A*, *SlARF8A*, and *SlARF24* was amplified by sequence-specific primers (Supplementary Table S2), respectively. Since *SlARF8A* and *SlARF8B* are highly homologous, the fragment from *SlARF8A* was used to silence both *SlARF8A* and *SlARF8B*. All of the pTRV1, pTRV2, pTRV2-PDS, and pTRV2-host target genes were transformed into the *Agrobacterium tumefactions* strain GV3101 by electroporation. Cultures containing the pTRV1 and pTRV2 vectors were mixed in a 1:1 ratio, either individually or simultaneously (*pTRV2-PDS*, *pTRV2*, *pTRV2-SlARF6A*, *pTRV2-SlARF8A*, *pTRV2-SlARF24*, *pTRV2-SlARF6A/8A*, *pTRV2-SlARF6A/24*, *pTRV2-SlARF8A/24*, *pTRV2-SlARF6A/8A/24*). These were used to infect cotyledon of tomato *e* mutant plants before the emergence of true leaves. The infected plants were transferred to a growth chamber at 16 h day/8 h night cycle, 20–22°C and 50% RH. The phenotypes were analyzed 6–7 weeks after inoculation. The VIGS method was following the published protocol (Velasquez et al., 2009).

### Quantification of the Free IAA Using UFLC-ESI-MS/MS

The sample leaves were frozen in liquid nitrogen. Three replicates were prepared for each leaf sample. The biomass for each replicate was 0.1 g. Subsequently, IAA extraction was performed by ESI-MS/MS following the published protocol (Liu et al., 2012).

### Yeast One-Hybrid Assay (Y1H)

For yeast one-hybrid assay, the –1479 bp fragment (upstream from the start codon) from the *SlPIN1* (Solyc03g118740) promoter was amplified from Ailsa Craig genomic DNA and cloned into the pAbAi vector (Clontech). Recombinant plasmid pAbAi-SlPIN1 and pGADT7-SlARF were co-transformed into the yeast strain Y1HGold (Clontech) by small-scale yeast transformation method respectively. The transformants were plated on the SD/-Ura drop-out medium. Colonies were picked and diluted in sterile ddH_2_O to an OD600 of 0.5, and 3 μl of suspension was spotted on SD/-Ura/-Leu drop-out medium with or without AbA antibiotic at 30°C. Both pGAD-p53+p53-AbAi (positive control) and pGADT7+P1-AbAi (negative control) were included.

### Transient Expression in Tobacco Leaves

The full-length SlARFs ORF were amplified and cloned into the effector vector, pGreen II 62-SK. A –1479 bp fragment (upstream from the start codon) from the *SlPIN1* promoter was amplified and cloned into the reporter vector, pGreen II 0800-LUC. Both the effector and reporter vector were respectively co-transformed into the *Agrobacterium tumefactions* strain GV3101 cells with the pSoup vector, then infiltrated into *N. benthamiana* young leaves and incubated 72 h in the dark. LUC and REN were analyzed using the dual luciferase assay reagents (Promega) with an Infinite M200 (Tecan). All primers used in this analysis are listed in Supplementary Table S2.

## Results

### SlIAA9 Interacts With Multiple SlARF Proteins

**FIGURE 1:**
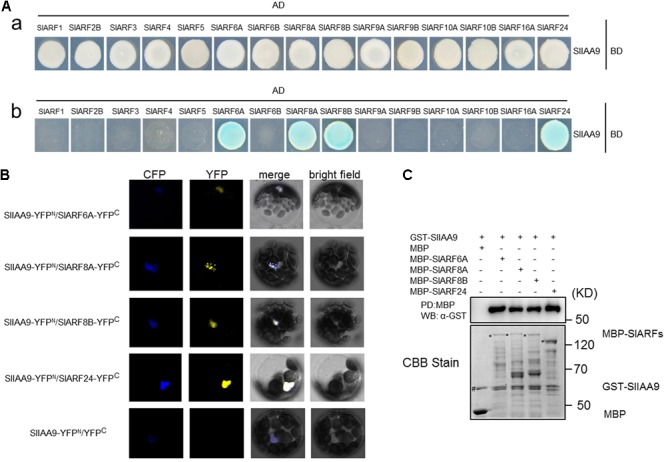
Interaction of SlIAA9 and the cloned SlARF proteins *in vivo* and *in vitro*. **(A)** The yeast cells grown on SD/-Leu/-Trp **(a)** and SD/-Ade/-His/-Leu/-Trp + 40 ug ml^-1^ X-gal **(b)**. **(B)** SlIAA9 interacts with SlARF6A, SlARF8A, SlARF8B, and SlARF24 in *Arabidopsis thaliana* cell culture protoplasts. YFP fluorescence was detected when SlIAA9-YFP^N^ was coexpressed with SlARF6A-YFP^C^, SlARF8A-YFP^C^, SlARF8B-YFP^C^, and SlARF24-YFP^C^, respectively. **(C)** GST-fused SlIAA9 proteins were incubated with MBP, MBP-SlARF6A, MBP-SlARF8A, MBP-SlARF8B or MBP-SlARF24 beads (PD:MBP). The incubated beads were washed and pelleted for immunoblot analysis with α-GST antibody. The protein inputs are indicated by Coomassie Brilliant Blue (CBB) staining.

In order to dissect the mechanism of *SlIAA9* regulating leaf shape development in tomato, a yeast two-hybrid (Y2H) screening was performed to identify the SlIAA9 interacting proteins from tomato cDNA Y2H library. After several screens, SlARF24 was screened out (Supplementary Table S3). To identify more SlARFs that may participate in this pathway, the full-length coding sequences of 15 *SlARFs*, including the candidate *SlARF24* previously identified, were isolated and inserted into the yeast two hybrid vector pGADT7, including *SlARF1*, *SlARF2B*, *SlARF3*, *SlARF4*, *SlARF5*, *SlARF6A*, *SlARF6B*, *SlARF8A*, *SlARF8B*, *SlARF9A*, *SlARF9B*, *SlARF10A*, *SlARF10B*, and *SlARF16A*. The recombinant plasmid pGBKT7-SlIAA9 and pGADT7-SlARFs were co-transformed into the yeast, respectively. Specifically, we observed that yeast cells containing pGBKT7-SlIAA9 mated with pGADT7-SlARF6A, pGADT7-SlARF8A, pGADT7-SlARF8B, and pGADT7-SlARF24 respectively to grow under selection conditions (**Figure 1A**). This result suggests that SlIAA9 may interact with SlARF6A, SlARF8A, SlARF8B, and SlARF24 in yeast cells.

Subsequently, the interactions between SlIAA9 and SlARF6A, SlARF8A, SlARF8B, and SlARF24 were examined using BiFC in *Arabidopsis* mesophyll protoplasts. We transformed the *Arabidopsis* Col-0 protoplasts with SlIAA9-YFP^N^/SlARF6A-YFP^C^_,_ SlIAA9-YFP^N^/SlARF8A-YFP^C^, SlIAA9-YFP^N^/SlARF8B-YFP^C^, SlIAA9-YFP^N^/SlARF24-YFP^C^, and SlIAA9-YFP^N^/YFP^C^. Strong YFP fluorescence signal was detected throughout the nucleus when SlIAA9-YFP^N^ was co-expressed with SlARF6A-YFP^C^, SlARF8A-YFP^C^, SlARF8B-YFP^C^_,_ and SlARF24-YFP^C^, whereas no fluorescence was detected in the control cells (**Figure 1B**). These results indicate that SlIAA9 might interact with multiple SlARF proteins *in vivo*.

In order to further confirm that the SlIAA9 protein could directly interact with SlARF6A, SlARF8A, SlARF8B, and SlARF24 proteins, a pull-down assay was performed. In this experiment, SlARF6A, SlARF8A, SlARF8B, or SlARF24 fused to maltose binding protein (MBP) immobilized on amylose-agarose beads were used as bait against GST-SlIAA9 fusion proteins. As shown in **Figure 1C**, GST-SlIAA9 could be pulled down by MBP-SlARF6A, MBP-SlARF8A, MBP-SlARF8B, as well as MBP-SlARF24, but not by MBP alone, demonstrating that SlARF6A, SlARF8A, SlARF8B, and SlARF24 proteins physically interact with SlIAA9 *in vitro*.

### The *e* Mutation Alters the Expression Patterns of Multiple *SlARFs*

A previous study showed that the basal gene expression of *SlIAA9* was high in roots, leaves, flowers, and fruits (Wang et al., 2005). For comparative purposes, qRT-PCR was performed to investigate the expression levels of *SlIAA9* in wild type root, stem, leaf, flower, and fruit tissue (Supplementary Figure S1). These results indicated that *SlIAA9* expressed in all tissues, but exhibits lower expression at the mature green (MG) stage in fruit tissue.

**FIGURE 2:**
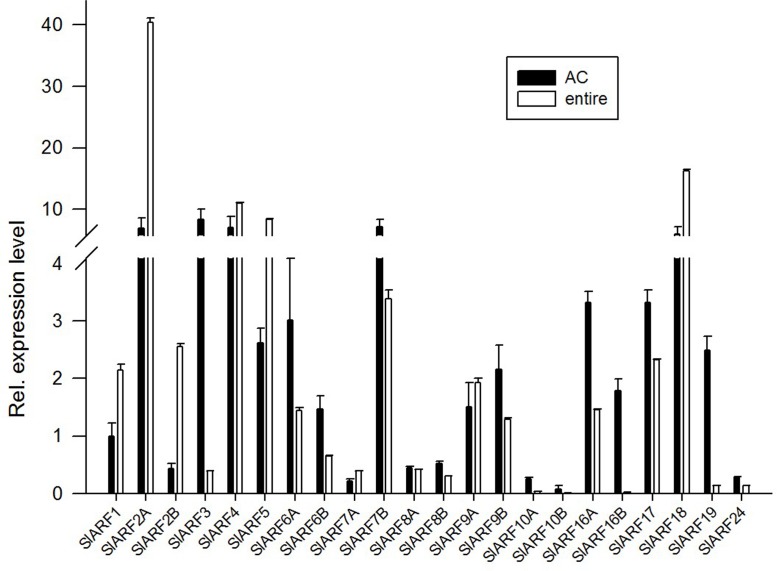
The expression patterns of Multiple *SlARFs* were altered by *e* mutation. The expression of *SlARF* genes family in wild type (compound leaves material) and *e* mutant (simple leaves material) leaves. The relative expression was referred using the level of *SlARF1* in the wild type control plant as calibrator. Standard errors (SE) are shown (*n* = 3).

We analyzed the expression levels of the *SlARF* genes family in wild type (compound leaves) and *e* mutant (simple leaves) leaves. The expression levels of *SlARF1*, *SlARF2A*, *SlARF2B*, *SlARF5*, and *SlARF18* in leaves of the *e* mutant were induced 2.14-, 5.79-, 5.90-, 3.19-, and 2.73-fold more than that of those in wild type leaves. While the *SlARF3*, *SlARF6A*, *SlARF6B*, *SlARF7B*, *SlARF10A*, *SlARF10B*, *SlARF16A*, *SlARF16B*, *SlARF19*, and *SlARF24* showed decreased expression corresponding to 0.05-, 0.48-, 0.45-, 0.47-, 0.15-, 0.15-, 0.44-, 0.01-, 0.05-, and 0.48-fold, respectively. Other *SlARFs* showed no significant difference (**Figure 2**). These results establish that multiple *SlARFs* are regulated in the absence of SlIAA9 in the auxin signaling model. This is particularly indicated for the *SlARF16B* gene, which might have a unique and significant function in this pathway. In addition, the expression levels of *SlARF6A*, *SlARF8A*, *SlARF8B*, and *SlARF24* displayed varying degrees of attenuation in *e* mutant plants compared to wild type plants, supporting the hypothesis that the SlARF6A, SlARF8A, SlARF8B, and SlARF24 interaction with SlIAA9 has compromised biological function in *e* mutant plants.

### Diminished Expression of Multiple *SlARFs* Can Rescue the Leaf Phenotype of Tomato *e* Mutation

**FIGURE 3:**
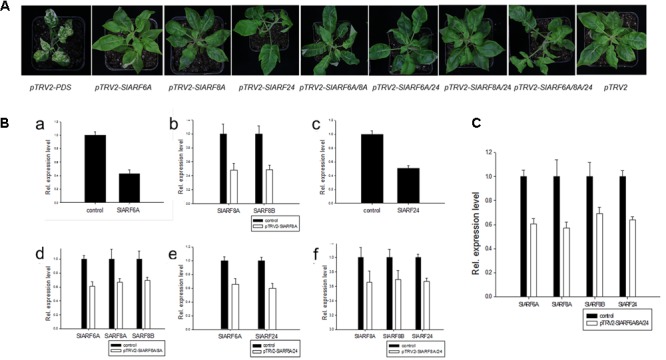
TRV-mediated virus-induced gene silencing (VIGS) of candidate *SlARF* genes in tomato *e* mutation leaves. SlARF8A and SlARF8B shared the same gene fragment in the VIGS vector. **(A)** Photographs of silencing tomato *e* mutation. **(B)** The candidate *SlARF* genes expression of injected with *pTRV2-SlARF6A* **(a)**, *pTRV2-SlARF8A* **(b)**, *pTRV2-SlARF24* **(c)**, *pTRV2-SlARF6A/8A* **(d)**, *pTRV2-SlARF6A/24* **(e)**, *pTRV2-SlARF8A/24* **(f)** cultures, respectively. **(C)** The *SlARF* genes expression of injected with *pTRV2-SlARF6A/8A/24* culture. The expression of a specific *SlARF* was compared with the level in *e* mutant plant agroinfiltrated with empty vector (control sample). Standard errors (SE) are shown (*n* = 3).

**FIGURE 4:**
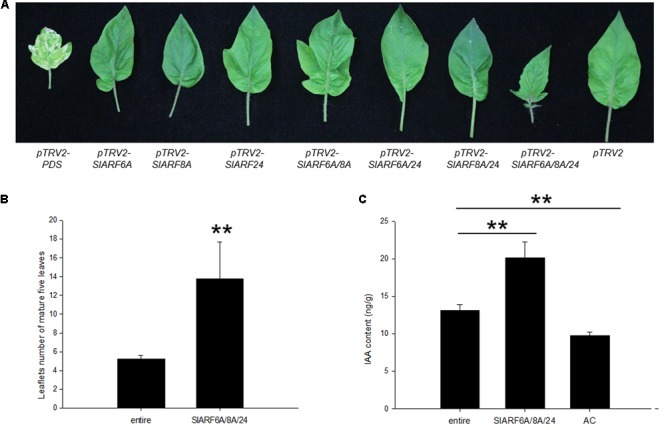
Leaves phenotypes and the free IAA contents of silencing of candidate *SlARF* genes in tomato *e* mutation. **(A)** The mature fifth leaves of the indicated phenotypes. **(B)** Quantification of the total leaflets number of the mature first five leaves (*n*= 5). **(C)** The IAA contents in *e* mutant and silencing of all the four *SlARF* genes materials. Standard errors (SE) are shown (*n* = 3). ^∗^*P* < 0.05, ^∗∗^*P* < 0.01.

The genetic mechanism by which *SlIAA9* regulates leaf shape development in tomato was examined by silencing *SlARFs* in the *e* mutant background. Using TRV-mediated VIGS we carried out a functional characterization assay of the four candidate *SlARF* genes identified through the previous expressional analysis. SlARF8A and SlARF8B share 82% amino acid identity (Supplementary Figure S2), therefore both genes were silenced in one VIGS construct. There was no detectable change in leaf shape after individually silencing *pTRV2-SlARF6A*, *pTRV2-SlARF8A*, and *pTRV2-SlARF24* (**Figure 3A**). Gene expression analysis showed that the mRNA levels of *SlARF6A*, *SlARF8A*, *SlARF8B*, and *SlARF24* were reduced to approximately 43%, 48%, 49%, and 51% respectively compared to the empty vector control (**Figure 3B**). Functional redundancies among ARF proteins have been described in *Cucumis sativus*, *A. thaliana*, and *S. lycopersicum* (Okushima et al., 2005; Liu and Hu, 2013; Hao et al., 2015). In accordance with this redundancy, we inoculated *pTRV2-SlARF6A*, *pTRV2-SlARF8A*, and *pTRV2-SlARF24* constructs into *e* mutant plants using double or triple co-cultures of *Agrobacterium*. Interestingly, 80%, 73%, and 77% of the *e* mutant plants infiltrated with *Agrobacterium* triple co-cultures expressing *pTRV2-SlARF6A/8A/24* were partially converted to compound leaves in three replicate experiments (**Table 1**). Meanwhile, the mRNA levels of *SlARF6A*, *SlARF8A*, *SlARF8B*, and *SlARF24* in the *e* mutant plants inoculated with the *Agrobacterium* co-cultures of *pTRV2-SlARF6A/8A/24* were reduced to approximately 60%, 57%, 69%, and 64% compared with the empty vector control, respectively (**Figure 3C**). Furthermore, the gene expression of other members of the *SlARF* family were not significantly changed after silencing the four candidate *SlARFs* (Supplementary Figure S3). Moreover, the fifth leaves of silencing of candidate *SlARF* genes in tomato *e* mutation were observed, the results presented that *e* mutant plants inoculated with *pTRV2-SlARF6A/8A/24* triple combination cultures were partially converted to wild-type compound leaves, which generating more leaflets (**Figure 4A**). The total leaflets on the mature first five leaves of the *pTRV2-SlARF6A/8A/24* inoculated plants were significantly increased compared to the *e* mutant plants (**Figure 4B**). The double co-cultures *pTRV2-SlARF6A/8A*, *pTRV2-SlARF6A/24*, and *pTRV2-SlARF8A/24* could not restore the development of compound leaves (**Figure 4A** and **Table 1**) despite that the expression analysis revealed the target genes were down-regulated (**Figure 3B**). These results illustrated that simultaneously silencing of these four genes could restore the compound leaf shape in *e* mutant plants, and suggested functional redundancies among SlARF proteins in regulating tomato leaf shape development.

**Table 1:** Statistical information describing the TRV-mediated virus-induced gene silencing (VIGS) of genes in *e* mutant plants.

Bacterial culture	Experimental times	No. of inoculation *e* mutant plants	No. of compound leaves plants	The percentage of compound leaves plants (%)
*pTRV2-PDS*	1	6	0	a
	2	8	0	a
	3	9	0	a
*pTRV2*	1	6	0	0
	2	8	0	0
	3	9	0	0
*pTRV2-SlARF6A*	1	15	1	7
	2	10	0	0
	3	13	0	0
*pTRV2-SlARF8A*	1	15	1	7
	2	10	1	10
	3	13	0	0
*pTRV2-SlARF24*	1	15	1	7
	2	10	1	10
	3	13	0	0
*pTRV2-SlARF6A/8A*	1	10	0	0
	2	15	1	7
	3	13	0	0
*pTRV2-SlARF6A/24*	1	10	1	10
	2	15	2	13
	3	13	1	8
*pTRV2-SlARF8A/24*	1	10	0	0
	2	15	1	7
	3	13	0	0
*pTRV2-SlARF6A/8A/24*	1	5	4	80
	2	30	22	73
	3	35	27	77

^*a*^Indicates that the percentage of albino plants injected with *pTRV2-PDS* cultures reached to 100%.

To analyze whether these phenotypic changes are regulated by auxin, the free IAA levels were quantified using UFLC-ESI-MS/MS. IAA levels of young leaves that had all four *SlARF*s silenced reached 20.14 ng/g, which were significantly higher than the *e* mutant plants (13.14 ng/g). Interestingly, the concentration of IAA in *e* mutant was observed to have a higher basal level than the wild type control plants (9.74 ng/g) (**Figure 4C**). This result suggested that the development of compound leaves inoculated with *pTRV2-SlARF6A/8A/24* were induced by auxin through promoting auxin response.

### Downregulation of Multiple *SlARFs* Can Rescue the Expression of Leaf Shape Determining Genes

**FIGURE 5:**
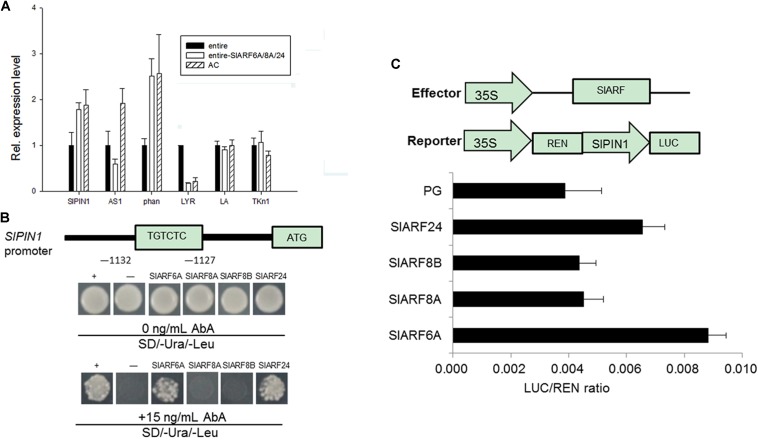
Quantitative real-time PCR analysis of leaf shape related genes and SlARF6A and SlARF24 bind to the *SlPIN1* promoter. **(A)** The expression of *SlPIN1*, *AS1*, and *phan* in the leaves of *e* mutant, silencing of all the four *SlARF* genes materials and wild type plants. **(B)** Yeast-one hybrid (Y1H) assay of SlARF6A and SlARF24 binding to *SlPIN1* promoter. The yeast cells grown on SD/-Ura/-Leu and SD/-Ura/-Leu + 15 ng ml^-1^ AbA. **(C)** Transcription activity assay in tobacco to examine the interaction between candidate SlARFs and *SlPIN1* promoter. The schematic diagrams constructs used for the dual LUC assay; REN (*Renilla luciferase*), a control for activity normalization; PG, the empty vector of pGreen II 62-SK. Standard errors (SE) are shown (*n* = 3).

The leaf growth and development in tomato is likely driven by the leaf shape related genes (Hareven et al., 1996; David-Schwartz et al., 2009; Illing et al., 2009; Yanai et al., 2011; Pinon et al., 2013). Subsequently, the transcript levels of these leaf shape related genes in tomato were evaluated through qRT-PCR. The *SlPIN1* and *phan* expression in *pTRV2-SlARF6A/8AB/24* silenced plants was induced 1.8- and 2.5-fold compared to *e* mutant plants, respectively. In contrast, the expression level of *LYR* was reduced by 4.6-fold compared to *e* mutant plants (**Figure 5A**). Thus, after silencing the four *SlARFs* in *e* mutant, the expression of those genes were reverted back to wild type level. We hypothesize that SlARF proteins may regulate leaf shape development by regulating the expression of *SlPIN1*.

### SlARF6A and SlARF24 Bind to the *SlPIN1* Promoter

To investigate the relationship between the candidate SlARFs proteins and *SlPIN1* promoter, a fragment of the *SlPIN1* promoter 1,479 bp upstream from the start codon was used in an Y1H assay (**Figure 5B**). Y1H results demonstrated that this fragment could interact with SlARF6A and SlARF24 protein, confirming that SlARF6A and SlARF24 protein recognize the *cis*-element in the *SlPIN1* promoter in yeast.

To determine whether SlARF6A and SlARF24 function as activator or repressor, we used a LUC (dual luciferase) assay to test how SlARF6A and SlARF24 interact with the *SlPIN1* promoter. The same fragment of *SlPIN1* used in the Y1H assay was introduced into the pGreen II 0800-LUC vector to generate the reporter construct (**Figure 5C**). The effector and reporter construct were transiently expressed in tobacco leaves and the relative LUC activity was determined. This result revealed that LUC activity was 2.29- and 1.69-fold higher in the presence of the SlARF6A and SlARF24 effector and reporter construct than in the negative control (**Figure 5C**), implying that both SlARF6A and SlARF24 may function as a transcriptional activator. This result revealed that the *cis*-element from the *SlPIN1* promoter was bound by SlARF6A and SlARF24.

## Discussion

### SlARF Proteins Regulate Tomato Leaf Shape in a Functionally Redundant Manner

Over the past 10 years, *SlIAA9* has been shown to be involved in fruit development, leaf morphogenesis, and fruit parthenocarpy in *A. thaliana* and *S. lycopersicum* (Wang et al., 2005, 2009; Goetz et al., 2007). In this study, we aimed to identify interacting partners of SlIAA9. The initial Y2H assay identified several candidates, including Aux/IAA proteins, ubiquitin related proteins, gibberellin beta-hydroxylase protein, MADs box interactor-like protein, and SlARF24 (Supplementary Table S3). More SlARFs (SlARF6A, SlARF8A, SlARF8B, and SlARF24) were further found to interact with SlIAA9 *in vivo* and *in vitro* (**Figure 1**), indicating that other SlARFs may play redundant roles with SlARF24 in regulating leaf development. SlIAA9 and miR160-targeted ARFs SlARF10A, SlARF10B, or SlARF17, appear to partially act in a functionally redundant manner, but remain necessary for local inhibition of lamina growth between initiating leaflets (Ben-Gera et al., 2016). However, the elucidation of the molecular mechanism on how these functional redundancies among SlARF proteins regulate tomato leaf shape has been hampered by complexity of the protein *in planta*. Understanding the mechanisms involved in leaf shape development in tomato can provide new insights into understanding these same mechanisms in other species such as *A. thaliana*, *Glycine max*, and *C. hirsuta*.

SlARFs may regulate leaf morphology through binding to the promoter of *SlPIN1* or other auxin-responsive genes in the absence of SlIAA9 (**Figure 5**). SlARF8 and SlIAA9 proteins, together with another unknown protein, may form a regulatory complex to control fruiting and growth, offering a possible explanation for the role of SlIAA9 in parthenocarpy (Goetz et al., 2007). *SlARF6* and *SlARF8* also play conserved roles in regulating development and growth of flower and vegetable organs in dicots (Liu et al., 2014). The *Osarf24-1* mutant presents reduced sensitivity to aberrant auxin signaling and auxin-deficient phenotypes (Sakamoto, 2013). Here, we firstly found that SlARF6A, SlARF8A, SlARF8B, and SlARF24 could regulate tomato leaf development in a redundant manner.

### *SlARF* Genes Play Distinct and Vital Roles in the Auxin Signaling Model

It has been illustrated that several *SlARF* genes might serve unique functions in tomato development. SlARF2A functions in the regulation of tomato fruit ripening as a recognized auxin signaling component (Breitel et al., 2016). Down-regulation of *SlARF4* results in a dark-green fruit phenotype with increased chloroplasts densities (Jones et al., 2002). Furthermore, SlARF4 involves in the control of sugar metabolism during fruit development in tomato (Sagar et al., 2013). Both auxin and gibberellin responses are modulated by SlARF7 during fruit formation and development in tomato (De Jong et al., 2011). Compared with wild type fruits, the fruits of *SlARF7*-RNAi transgenic lines presented seedless, heart-shaped, and thick pericarp phenotypes in tomato (De Jong et al., 2009). Cell division is negatively regulated by *SlARF9* during early fruit development in tomato (De Jong et al., 2015). Primexine formation is modulated by ARF17, which is crucial for pollen wall patterning, partially through regulation of *CalS5* gene expression in *Arabidopsis* (Yang et al., 2013). A 165-bp deletion in *ARF18* gene simultaneously affects silique length and seed weight in polyploid rapeseed (Liu et al., 2015).

Moreover, there are functional redundancies among ARF proteins in *Cucumis sativus*, *A. thaliana*, and *S. lycopersicum* (Okushima et al., 2005; Liu and Hu, 2013; Hao et al., 2015). A constitutive expression pattern was exhibited in almost all of the *ARF* genes in cucumber (Liu and Hu, 2013). In *A. thaliana*, *arf7 arf19* double mutant presented an obvious auxin-related phenotype that were not detectable in the single mutant, suggesting that there are functional redundancies between ARF7 and ARF19 proteins (Okushima et al., 2005). Simultaneous silencing of *SlARF2A* and *SlARF2B* genes leaded to severe ripening inhibition, clarifying a functional redundancies between SlARF2A and SlARF2B proteins (Hao et al., 2015). Our VIGS results provided evidence that functional redundancies among SlARF proteins resulted in the change from a simple leaf to a complex one in tomato *e* mutant plants (**Figure 3**). Due to the far evolutionary relationship among candidate SlARFs, clarifying there are functional compensation among the candidate SlARFs. It should be noted that this study has only examined the function of *SlARF6A*, *SlARF8A*, *SlARF8B*, and *SlARF24*, because only 15 tomato *SlARF* genes were isolated from the full-length cDNA sequences out of the 22 *SlARF* genes family. It has been reported that SlARF17 and SlIAA9 do not interact in yeast (Ben-Gera et al., 2016). There are still another six *SlARF* genes that have not yet been characterized by us. Our result showed that the expression levels of *SlARF16B* were 0.01-fold less in leaves of the *e* mutant compared to those in wild type. A previous study describes that the *Pto-ARF16* was affected by Pto-miR160a associated with tree growth and wood property traits in *Populus tomentosa* (Tian et al., 2016). In *A. thaliana*, ARF10 and ARF16 were targeted by miR160 to control the formation of root cap cell, and miR160-uncoupled production of ARF16 reflected pleiotropic effects (Wang et al., 2005). Thus, we hypothesize that *SlARF16B* is regulated in the absence of SlIAA9 in the auxin signaling model, which needs to further evaluated with additional experiments. Consequently, the *e* mutation likely alters the expression patterns of other *SlARF* genes through this mechanism. The hetero-dimerization between Aux/IAA and ARF proteins likely able to play unique cellular functions (Piya et al., 2014). However, how the SlIAA9-SlARFs complex functions during tomato leaf development is still not yet completely resolved.

### A Proposed Model of SlIAA9 Complex in the Control of Tomato Leaf Forms

We chose several leaf shape related genes in *A. thaliana* and *S. lycopersicum* to evaluate whether these genes could regulate the development of leaf shape. As a result, the expression levels of *SlPIN1*, *phan*, and *LYR* in leaves having *SlARF6A*, *SlARF8A*, *SlARF8B*, and *SlARF24* simultaneously silenced through VIGS were restored to levels similar to those in wild-type plants (**Figure 5A**). The IAA levels in leaves having those same four *SlARFs* silenced were significantly increased compared to the control (**Figure 4C**). Auxin acts as a positional cue during leaf organogenesis, and auxin efflux carrier PIN1 is one of the main contributors to auxin localization (Reinhardt et al., 2003; Cheng et al., 2007). PIN1 localizes on the periphery of apical meristems directing auxin to convergence points, where auxin maxima is formed, subsequently auxin becomes directed subepidermally at the leaf initiation site to regulate leaf development (Heisler et al., 2005; Martinez et al., 2016). Genetic analyses have also demonstrated that PIN1 is required for leaflet initiation in compound leaves (Barkoulas et al., 2008). Accordingly, the *cis*-element of *SlPIN1* was used for deep analysis. The *cis*-elements from *SlPIN1* promoter was recognized and bound specifically by SlARF6A and SlARF24 in yeast and plants (**Figure 5**). Here, we propose that SlARF6A and SlARF24 may regulate leaf growth and development through direct binding to the *SlPIN1* promoter. However, the effects of enhanced *SlPIN1* transcription still needs to be further evaluated. This enhanced transcription may result to increased expression of SlPIN1 protein, changed SlPIN1 protein modification, or shift the location of SlPIN1.

**FIGURE 6:**
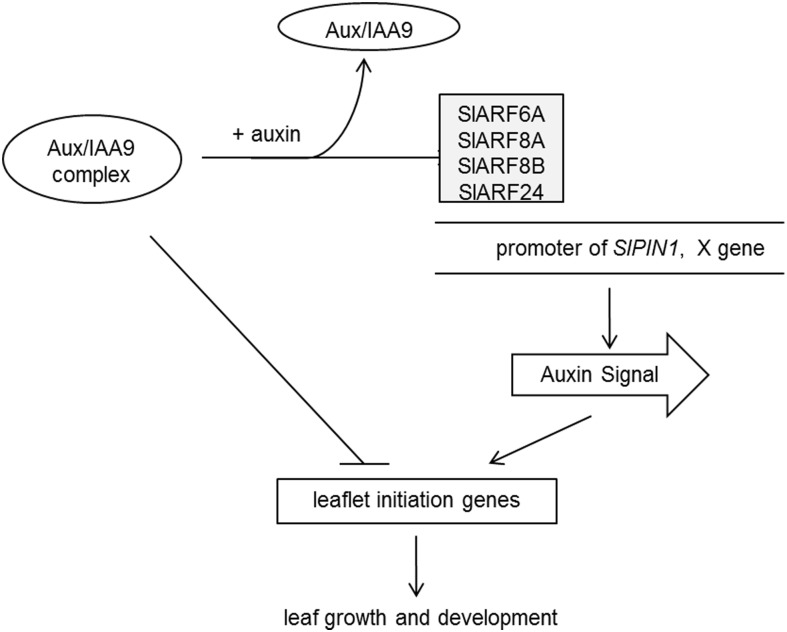
A proposed model of SlIAA9 complex in the control of tomato leaf forms. SlIAA9 and SlARF6A, SlARF8A, SlARF8B, and SlARF24 proteins, form a regulatory complex, which can directly block leaflet initiation genes or act indirectly by preventing SlARFs from functioning as transcriptional activators. In the absence of SlIAA9, SlARFs may regulate leaf growth and development through direct binding to the promoter of *SlPIN1* or other auxin-responsive genes, which promoted auxin response.

Our data provide an insight to suggest that SlARF proteins work with *SlIAA9* in a functionally redundant manner to dictate leaf shape. We propose that SlIAA9 interacts with multiple SlARF proteins to promote the formation of a regulatory complex which can directly block leaflet initiation genes. We also assume that this complex may act indirectly by preventing SlARFs from functioning as transcription activators. In the absence of SlIAA9, SlARFs may regulate leaf growth and development through direct binding to the promoter of *SlPIN1* or unknown X genes induced by auxin (**Figure 6**). Future studies will be directed to dissect the relationship between SlIAA9 and SlARF6A, SlARF8A, SlARF8B, and SlARF24. We also intend to clone other *SlARF* genes to ascertain whether SlIAA9 has additional interactors with unique biological functions.

## Conclusion

In conclusion, this study posits a proposed molecular mechanism of SlIAA9 complex in the control of tomato leaf forms (**Figure 6**). Our results firstly demonstrate that SlARF6A, SlARF8A, SlARF8B, and SlARF24 directly interact with SlIAA9, and the simple leaves of the *e* mutant are partially converted to wild-type compound leaves by silencing of all the four *SlARFs*. Meanwhile, SlARF6A and SlARF24 bind to the *SlPIN1* promoter to regulate genes expression involved in leaflet initiation. Further studies are still needed to explore the underlying mechanism of SlARF proteins in modulating tomato leaf shape.

## Author Contributions

LW designed and performed the experiments, data analysis, and drafted this manuscript. JZ supervised the experiments and revised the manuscript. JZ and ZT designed all the experiments.

## Conflict of Interest Statement

The authors declare that the research was conducted in the absence of any commercial or financial relationships that could be construed as a potential conflict of interest.

## ABBREVIATIONS

ARF: auxin response factor
IAA: indole-3-acetic acid
LUC: firefly luciferase
qRT-PCR: quantitative real-time PCR
REN: renilla luciferase
VIGS: virus-induced gene silencing
YFP: yellow fluorescent protein
