# Supplementary figures and images for: Functional Dissection of Auxin Response Factors in Regulating Tomato Leaf Shape Development
Source: Front Plant Sci. 2018 Jul 4;9:957. doi: 10.3389/fpls.2018.00957 (PMC6040142; doi:10.3389/fpls.2018.00957)

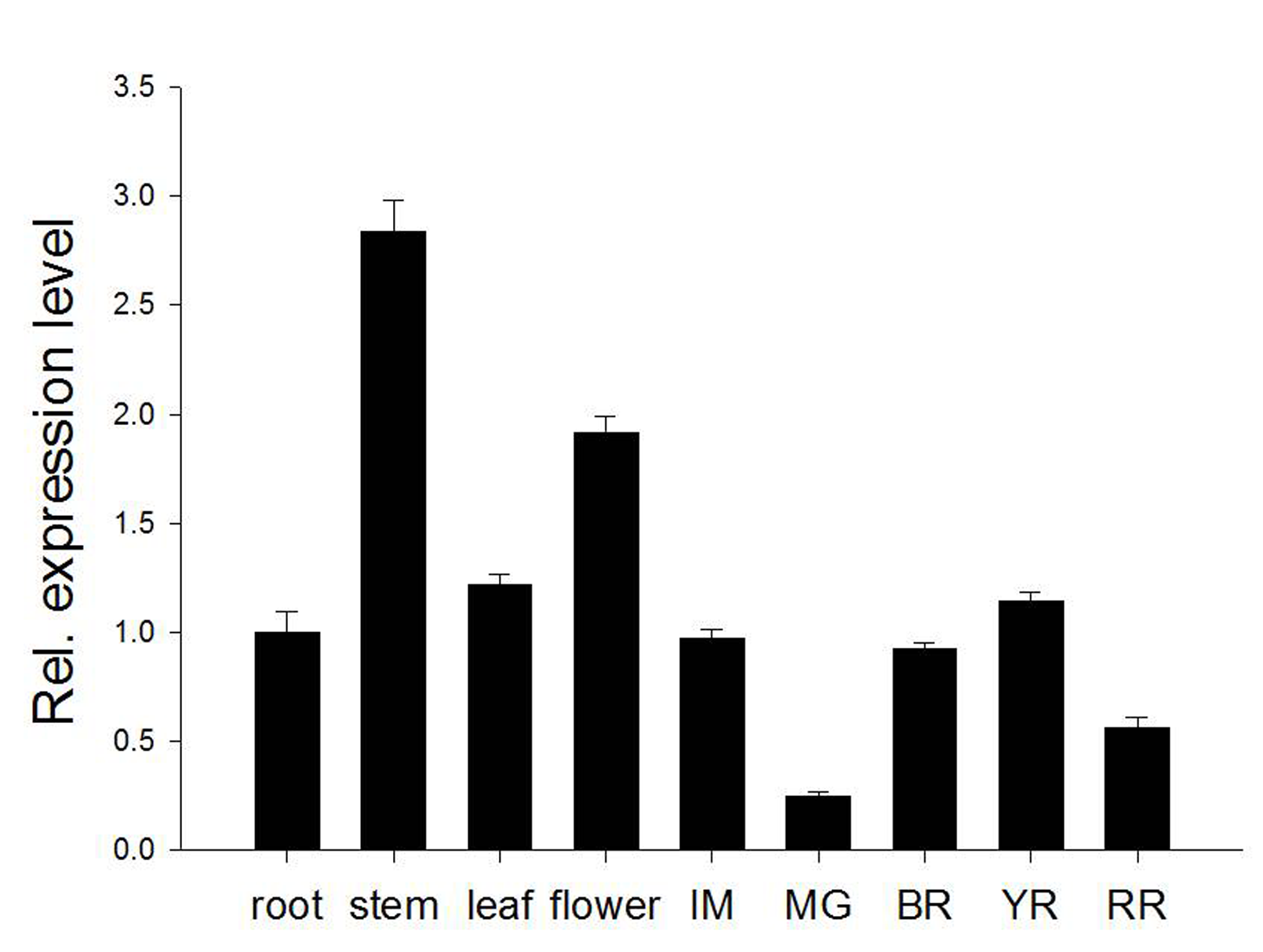

Supplement: Supplementary file 1 [file Image_1.TIF]

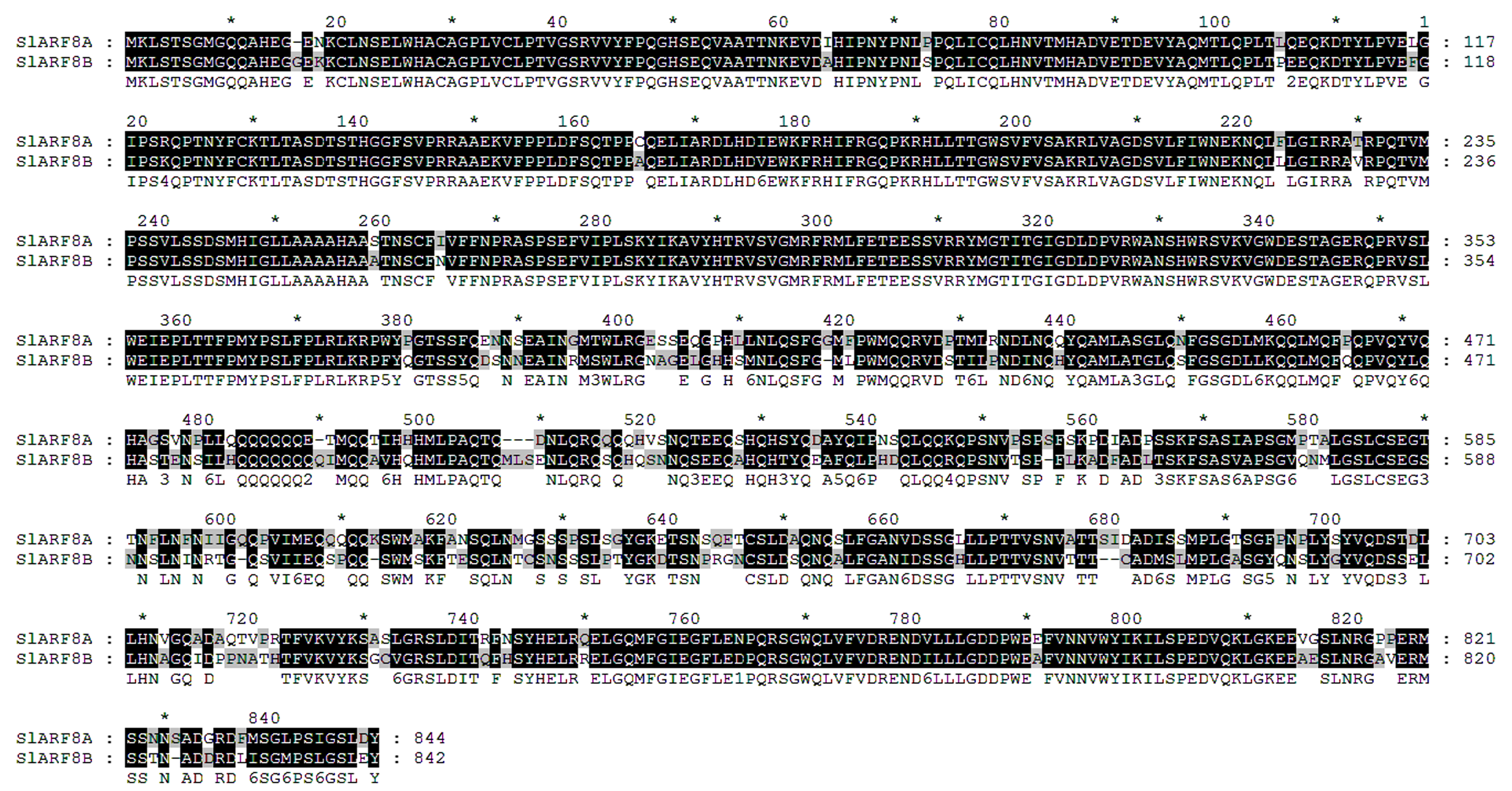

Supplement: Supplementary file 2 [file Image_2.TIF]

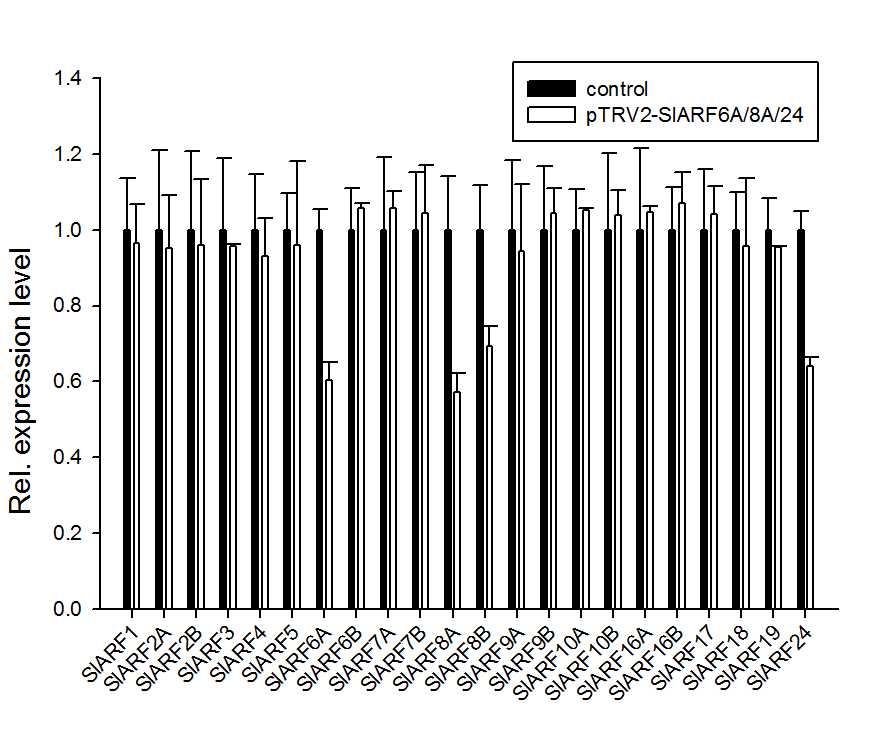

Supplement: Supplementary file 3 [file Image_3.TIF]
