# Supplementary material for: Functional Dissection of Auxin Response Factors in Regulating Tomato Leaf Shape Development
Source: Front Plant Sci. 2018 Jul 4;9:957. doi: 10.3389/fpls.2018.00957 (PMC6040142; doi:10.3389/fpls.2018.00957)
Supplement: Supplementary file 4 [file Table_1.DOCX]

| Generic name | Alias |
| --- | --- |
| SlARF1 | Solyc01g103050 |
| SlARF2A | Solyc03g118290 |
| SlARF2B | Solyc12g042070 |
| SlARF3 | Solyc02g077560 |
| SlARF4 | Solyc11g069190 |
| SlARF5 | Solyc04g081240 |
| SlARF6A | Solyc12g006340(Nter);Solyc00g196060(Cter) |
| SlARF6B | Solyc07g043620 |
| SlARF7A | Solyc07g016180 |
| SlARF7B | Solyc05g047460 |
| SlARF8A | Solyc03g031970 |
| SlARF8B | Solyc02g037530 |
| SlARF9A | Solyc08g082630 |
| SlARF9B | Solyc08g008380 |
| SlARF10A | Solyc11g069500 |
| SlARF10B | Solyc06g075150 |
| SlARF16A | Solyc09g007810 |
| SlARF16B | Solyc10g086130 |
| SlARF17 | Solyc11g013480(Nter);Solyc11g013470(Cter) |
| SlARF18 | Solyc01g096070 |
| SlARF19 | Solyc07g042260 |
| SlARF24 | Solyc05g056040 |
