# Supplementary material for: Functional Dissection of Auxin Response Factors in Regulating Tomato Leaf Shape Development
Source: Front Plant Sci. 2018 Jul 4;9:957. doi: 10.3389/fpls.2018.00957 (PMC6040142; doi:10.3389/fpls.2018.00957)
Supplement: Supplementary file 5 [file Table_2.DOCX]

Gene Purpose sequence (5’-3’)

| *SlIAA9* | BiFC | Forward（BamHI）TGGCGCGCCACTAGTGGATCCATGTCTCCGCCGCTCCTTGG  Reverse（SalI） AGCGGTACCCTCGAGGTCGACATTTCTGCTCCGACACTT |
| --- | --- | --- |
| *SlARF6A* | BiFC | Forward（BamHI） TGGCGCGCCACTAGTGGATCCATGGCAGCAGGGGAGAAGAA  Reverse（SalI） AGCGGTACCCTCGAGGTCGACGTACTCAAGTGACCCCAGGG |
| *SlARF8A* | BiFC | Forward（BamHI）TGGCGCGCCACTAGTGGATCCATGAAGCTTTCAACATCAGG  Reverse（SalI） AGCGGTACCCTCGAGGTCGACGTAATCAAGTGATCCTATAG |
| *SlARF8B* | BiFC | Forward（BamHI）TGGCGCGCCACTAGTGGATCCATGAAGCTTTCAACATCAGG  Reverse（SalI） AGCGGTACCCTCGAGGTCGACGTACTCCAGCGATCCAAGAG |
| *SlARF24* | BiFC | Forward（BamHI）TGGCGCGCCACTAGTGGATCCATGGTTGATCAATTGCGGTT  Reverse（SalI） AGCGGTACCCTCGAGGTCGACCAACAAACCCTCCAAGACAG |
| *SlIAA9* | pull-down | Forward（EcoRI）GTTCCGCGTGGATCCCCGGAATTCATGTCTCCGCCGCTCCTTGG  Reverse（XhoI） TCAGTCACGATGCGGCCGCTCGAGCTAATTTCTGCTCCGACACT |
| *SlARF6A* | pull-down | Forward（EcoRI）GAGGGAAGGATTTCAGAATTCATGGCAGCAGGGGAGAAGAA  Reverse（SalI） CAAGCTTGCCTGCAGGTCGACTTAGTACTCAAGTGACCCCA |
| *SlARF8A* | pull-down | Forward（EcoRI）GAGGGAAGGATTTCAGAATTCATGAAGCTTTCAACATCAGG  Reverse（SalI） CAAGCTTGCCTGCAGGTCGACTCAGTAATCAAGTGATCCTA |
| *SlARF8B* | pull-down | Forward（EcoRI）GAGGGAAGGATTTCAGAATTCATGAAGCTTTCAACATCAGG  Reverse（SalI） CAAGCTTGCCTGCAGGTCGACTCAGTACTCCAGCGATCCAA |
| *SlARF24* | pull-down | Forward（EcoRI）GAGGGAAGGATTTCAGAATTCATGGTTGATCAATTGCGGTT  Reverse（SalI） CAAGCTTGCCTGCAGGTCGACCTACAACAAACCCTCCAAGA |
| *SlARF6A* | VIGS | Forward（EcoRI）CTGTGAGTAAGGTTACCGAATTCCACCTGGACTGCCTTCATTT  Reverse（BamHI）CGCGTGAGCTCGGTACCGGATCCTCTAATGCTGCCATTGCTTG |
| *SlARF8A* | VIGS | Forward（EcoRI）CTGTGAGTAAGGTTACCGAATTCGACATGGTTGAGAGGGGAAA  Reverse（BamHI）CGCGTGAGCTCGGTACCGGATCCCAAACCGCTAGCCAGCATA |
| *SlARF24*  *SlPIN1*  *SlARF6A*  *SlARF8A*  *SlARF8B*  *SlARF24* | VIGS  LUC  LUC  LUC  LUC  LUC | Forward（EcoRI）CTGTGAGTAAGGTTACCGAATTCTTGATCAATTGCGGTTTTCA  Reverse（BamHI）CGCGTGAGCTCGGTACCGGATCCCGACAAAGGATCTTTGGAGGT  Forward（KpnI） CACTATAGGGCGAATTGGGTACCTGACACACTAATCTTAATTTGCT  Reverse（NcoI） TATGTTTTTGGCGTCTTCCATGGCTTTGCTATGTTCTTGATATACC  Forward（BamHI）GCCGCTCTAGAACTAGTGGATCCATGGCAGCAGGGGAGAAGAAAAG  Reverse（XhoI） TTGGTACCGGGCCCCCCCTCGAGTTAGTACTCAAGTGACCCCAGGG  Forward（BamHI）GCCGCTCTAGAACTAGTGGATCCATGAAGCTTTCAACATCAGGAAT  Reverse（XhoI） TTGGTACCGGGCCCCCCCTCGAGTCAGTAATCAAGTGATCCTATAG  Forward（BamHI）GCCGCTCTAGAACTAGTGGATCCATGAAGCTTTCAACATCAGGAAT  Reverse（XhoI） TTGGTACCGGGCCCCCCCTCGAGTCAGTACTCCAGCGATCCAAGAG  Forward（BamHI）GCCGCTCTAGAACTAGTGGATCCATGGTTGATCAATTGCGGTTTTC  Reverse（XhoI） TTGGTACCGGGCCCCCCCTCGAGCTACAACAAACCCTCCAAGACAG |
