# Supplementary material for: Functional Dissection of Auxin Response Factors in Regulating Tomato Leaf Shape Development
Source: Front Plant Sci. 2018 Jul 4;9:957. doi: 10.3389/fpls.2018.00957 (PMC6040142; doi:10.3389/fpls.2018.00957)
Supplement: Supplementary file 6 [file Table_3.DOCX]

| Locus name | Putative protein | Possible function |
| --- | --- | --- |
| Solyc06g069090.2.1 | 40S ribosomal protein | protein biosynthesis |
| Solyc10g080160.1.1 | 30s ribosomal proteins | protein biosynthesis |
| Solyc06g053840.2.1 | auxin-regulated IAA1 | auxin-regulated protein |
| Solyc06g053830.2.1 | auxin-regulated IAA7 | auxin-induced protein |
| Solyc04g076850.2.1 | entire | auxin protein, IAA9 |
| Solyc03g121060.2.1 | auxin-regulated IAA14 | phytochrome-associated protein1 |
| Solyc05g056040.2.1 | Auxin Response Factor 24 | auxin response factors |
| Solyc07g064130.1.1 | ubiquitin/UBQ | unknown |
| Solyc03g113100.2.1 | ubiquitin-conjugating enzyme E2 | protein degredation |
| Solyc05g015520.2.1 | F-box family protein | unknown |
| Solyc01g105660.2.1 | giberellin beta-hydroxylase | giberellin beta-hydroxylase |
| Solyc10g081180.1.1 | Glycine-rich protein7 | nucleic acid binding |
| Solyc11g012320.1.1 | ripening-related mRNA 13 | unknown |
| Unknown | late maturation | unknown |
| Solyc11g030390.1.1 | MADs box interactor-like | unknown |
| Solyc11g062130.1.1 | ADP/ATP translocator | transport |
| Solyc12g099080.1.1 | ADP-ribosylationfactor protein | ADP-ribosylation factor 1 |
| Solyc02g085950.2.1 | Glucose pyrophosphorylase subunit | rubisco, small subunit 3A/3C |
| Solyc07g032090.2.1 | IIF subunit beta | nucleic acid metabolism |
| Solyc03g063240.2.1 | FMN binding | root border cell-specific protein |
| Solyc02g064670.1.1 | unknown | ribosomal protein |
| Unknown | leaf trichomes 4-8 weeks old | unknown |
| Solyc07g064930.2.1 | protein grpE | unknown |
| Solyc02g084550.2.1 | chromosome10 DNAsequence | unknown |
| Solyc09g097850.1.1 | Cysteine proteinase inhibitor | unknown |
| Solyc04g081730.2.1 | unknown protein | unknown |
| Solyc03g063480.2.1 | unknown protein | unknown |
