# Supplementary material for: Functional Dissection of Auxin Response Factors in Regulating Tomato Leaf Shape Development
Source: Front Plant Sci. 2018 Jul 4;9:957. doi: 10.3389/fpls.2018.00957 (PMC6040142; doi:10.3389/fpls.2018.00957)
Supplement: Supplementary file 7 [file Data_Sheet_1.docx]

**FIGURE S1｜ Expression analysis of *SlIAA9* in wild type (AC) different organs.**

**FIGURE S2｜ Amino acid sequences alignments of SlARF8A and SlARF8B.**

**FIGURE S3｜ Expression analysis of other members of the *SlARFs* family in the *e* mutant silencing of the four candidate *SlARFs* and control materials.**

**TABLE S1｜ The *SlARF* genes family in tomato**

**TABLE S2｜ Primers used in this study**

**TABLE S3｜ The putative functions of cDNA inserted sequences in the pGADT7 vector**
